# Specific Micro RNA-Regulated TetR-KRAB Transcriptional Control of Transgene Expression in Viral Vector-Transduced Cells

**DOI:** 10.1371/journal.pone.0051952

**Published:** 2012-12-14

**Authors:** Virginie Pichard, Dominique Aubert, Sebastien Boni, Severine Battaglia, Dejana Ivacik, Tuan Huy Nguyen, Patrick Arbuthnot, Nicolas Ferry

**Affiliations:** 1 Institut National de la Santé et de la Recherche Médicale (INSERM) Unité Mixte de Recherche (UMR) 948, Nantes, France; 2 INSERM UMR 1089, Nantes, France; 3 INSERM UMR 957, Nantes, France; 4 INSERM UMR 1064, Nantes, France; 5 Antiviral Gene Therapy Research Unit, School of Pathology, University of Witwatersrand, Johannesburg, Afrique du Sud; University of Massachusetts Medical, United States of America

## Abstract

Precise control of transgene expression in a tissue-specific and temporally regulated manner is desirable for many basic and applied investigations gene therapy applications. This is important to regulate dose of transgene products and minimize unwanted effects. Previously described methods have employed tissue specific promoters, miRNA-based transgene silencing or tetR-KRAB-mediated suppression of transgene promoters. To improve on versatility of transgene expression control, we have developed expression systems that use combinations of a tetR-KRAB artificial transgene-repressor, endogenous miRNA silencing machinery and tissue specific promoters. Precise control of transgene expression was demonstrated in liver-, macrophage- and muscle-derived cells. Efficiency was also demonstrated in vivo in murine muscle. This multicomponent and modular regulatory system provides a robust and easily adaptable method for achieving regulated transgene expression in different tissue types. The improved precision of regulation will be useful for many gene therapy applications requiring specific spatiotemporal transgene regulation.

## Introduction

Tight spatial and temporal control of gene expression is a prerequisite for many basic investigations and therapeutic applications involving gene transfer. Unintended adverse effects may result from expression of a transgene in non-targeted cell types. For example, exogenous protein expression in antigen presenting cells may trigger an immune response against the transgene product. In other instances, such as the use of toxic genes to eliminate diseased cells, precise control of gene expression is important to ensure safety and avoid unwanted secondary effects. Unintended sequelae of gene transfer may thus result from expression in non-target tissues as well as from protracted high level expression of the transgene. Various strategies have been designed and tested to achieve specific and tightly regulated gene expression. Initially, accomplishing limited transgene expression was restricted to the use of tissue-specific promoters inserted into engineered cassettes. Although valuable results have been obtained using this approach [1], [2], these promoters are often weaker than ubiquitously active promoters. Moreover, leakiness of transcriptional regulatory elements often results in transgene expression in non-targeted cells [3], [4], [5].

Inducible systems have been developed in which expression of the transgene is controlled through administration of specific molecules. Many of the approaches are based on transactivation of artificial promoters by chimeric transcription factors. The widely used tetracycline (tet)-regulatable system was first described by Gossen and Bujard [6]. It is based on the fusion of the Tet repressor protein (TetR) with the VP16 transactivator of the Herpes simplex virus. The TetR from E.Coli Tn10 operon binds to the Tet operator DNA sequence (*tetO*) specifically. In the presence of tet, the TetR conformation changes and it no longer binds to *tetO* sequences. When fused to VP16, the TetR-VP16 chimeric protein is able to activate DNA transcription, provided that the *tetO* sequence is present in target DNA. The TetR-VP16 chimera is also sensitive to tet-induced conformational changes. In the presence of the drug, TetR-VP16 does not bind DNA and as a result transcription is inactivated. This system is known as the Tet-Off regulatable system. Mutagenesis in TetR allowed for isolation of mutant proteins, known as reverse TetR (rTetR) that bind DNA targets when the drug is present. Rather than binding to DNA in the absence of tet, rTetR only binds DNA in the presence of the drug. This mutant derivative was called the Tet-On regulatable system [7]. Using another strategy, some investigators sought to develop a tet regulatable system based on the fusion of TetR with a transcriptional repressor domain, the Krüppel-associated box protein (KRAB) [8]. KRAB is a 75 aa domain found in about one third of the several hundreds of human zinc finger proteins and is located in the amino terminal end of proteins that contain Krüppel-class zinc fingers in their carboxy termini. The KRAB domain acts by triggering formation of heterochromatin in the vicinity of its binding site and thus is able to shut off Polymerase I, II and III promoters. To induce heterochromatin formation, KRAB recruits KAP1 (KRAB-associated protein 1 also known as TRIM28 or KRIP1) which acts as a scaffold for the function of proteins such as heterochromatin protein 1, histone methyl transferase and histone deacetylase [9], [10]. It has been demonstrated that KRAB can mediate transcriptional repression over a range of tens of kilobases [11] and that this system is involved in the control of endogenous retroviruses in embryonic stem cells [12]. A chimeric transrepressor protein was engineered by fusing the KRAB domain of human Kox1 to the TetR. The resulting TetR-KRAB was able to bind to *tetO* sequences in the absence but not in the presence of Tet [8]. When *tetO* sequences were placed up to 3 kb upstream of a range of different polII promoters, silencing occurred in the absence of Tet when TetR-KRAB was expressed in the cells. KRAB is able to repress Pol III promoters and can be used to repress expression of Pol III-transcribed RNAs such as shRNAs that are used to silence endogenous genes [13], [14]. To harness these properties for gene therapy, a drug-inducible system was designed in which a lentiviral vector was used to transfer a Pol II cassette containing a reporter gene together with the TetR-KRAB coding sequence [14]. The two coding regions were separated by an internal ribosomal entry site (IRES) sequence. A *tetO* heptamer was inserted in the proviral LTR together with a H1 promoter driving expression of shRNAs. The results showed that conditional and reversible production of shRNAs could be achieved in vitro and in vivo after lentiviral gene transfer to organs or in transgenic animals.

In addition to use of tissue-specific and inducible promoters to control gene expression in target tissues, endogenous microRNAs (miRNAs) have recently been employed to regulate transgene expression [5], [15]. This approach takes advantage of variation in the abundance of specific miRNAs in different tissues to achieve differential RNA interference- (RNAi-) based gene silencing. Transgene cassettes are typically engineered to include multiple miRNA target sites in the 3′ untranslated region (UTR) of a mRNA. Interaction between a specific miRNA and the target can then be used to silence transgene expression efficiently. To refine the TetR-KRAB regulation system and achieve improved tissue-specific regulation of transgene expression, we have combined TetR-KRAB-mediated transcriptional suppression with miRNA-based tissue-specific control of gene expression. To add an additional layer of control, we included tissue-specific promoters. By combining the TetR-KRAB artificial transgene-repression system with miRNA silencing, efficient regulation of transgene expression was achieved. Importantly, transgene expression was dependent on abundance of miRs in specific tissues. We verified the functionality of this novel type of expression system in vivo and in vitro, in different target tissues.

## Materials and Methods

### Ethics Statement

All animals were handled in accordance with the Guide for the Care and Use of Laboratory Animals. The Ethics Committee of animals experiments of laboratory of the Pays de Loire (France) approved the protocol.

### In vivo Analysis

Balb/C mice (8–10 weeks of age) were injected with a total dose of 1×10^11^ viral genomes (vg) into each of the two tibialis anterior muscles. Doxycycline was administered in drinking water at a concentration of 2 g/litre supplemented with 4% sucrose. For whole-body in vivo analysis, anesthetized mice were imaged using NightOWL II LB 983 NC320 (Betthold technologies) imaging equipment. The fluorescence was quantified using the WINLIGHT software. Mice were anesthetized by isofluorane inhalation (3% v/v) and maintained under a 12 h light/dark illumination cycle with food and water *ad libitum*.

### Plasmid Design

We combined two different expression cassettes in the vector transfer plasmids. The first included a constitutively active promoter (PGK, phosphoglycerate kinase or CMV, cytomegalovirus) driving a TetR-KRAB sequence, which was linked to four tandem repeats of a target sequence designed to be perfectly complementary to miR-122 (TGG AGTGTGACAATGGTGTTTGTGT), miR-142.3p (TCCATAAAGTAGGAAACACTACA) and miR-133 (ACAGCTGGTTGAAGGGGACCAA). To terminate transcription, an unidirectional polyadenylation signal (pA) was inserted downstream of the cassette. The second cassette, in reverse orientation to the TetR-KRAB sequence, contained the *gfp* gene under control of a ubiquitously active CMV or liver -specific mTTR promoter. A *tetO* heptamer sequence was placed between the two cassettes. The entire construct was inserted into lentiviral or AAV backbones to generate the corresponding recombinant virus vectors. Function of this construct was compared to that of a previously described TetR-KRAB inducible system, which includes a bicistronic unit comprising a *gfp* gene under control of a CAG (CMV immediate enhancer/β-actin) promoter and the KRAB-based repressor (PLVCT) [14]. In this construct the *tetO* was inserted into the U3 region of the 3′ LTR.

### Production of Lentiviral Vectors

Lentiviral vector stocks were generated as previously described by using calcium phosphate-mediated transient transfection of 293T cells with a vector transfer plasmid, the packaging plasmid (psPAX2), and vesicular stomatitis virus G protein (VSVG) envelope protein-coding plasmid (pMD2G) [16]. Viral supernatant was collected daily for three days after transfection. Viruses were concentrated using high-speed centrifugation (71 490 × g) for 3 hours at 4°C. Pellets were resuspended and vector aliquots snap-frozen then stored at −80°C.

To determine the titers of GFP-transducing vectors, serial dilutions of vector stocks were used to transduce HeLa cells. DNA was extracted and vector titers were determined by real-time quantitative PCR (qPCR) as described below. The titers obtained were in the range of 10^6^ to10^7^ transducing particles/ml.

### Cell Culture and Infection

Huh7 hepatoma and 293T human epithelial kidney cells were grown in Dulbecco’s modified Eagle’s medium (DMEM) supplemented with 10% heat-inactivated fetal calf serum, 50 IU/ml penicillin, and 50 µg/ml streptomycin. NR8383 cells were cultured in Ham’s F12K medium containing 2 mM l-glutamine, 1.5 g/l sodium bicarbonate, 15% heat inactivated fetal bovine serum, 50 IU/ml penicillin and 50 µg/ml streptomycin [5]. C2C12 myoblasts were maintained at subconfluent densities in DMEM supplemented with 10% fetal bovine serum, 50 IU/ml penicillin, and 50 µg/ml streptomycin. Near-confluent cells were induced to differentiate with DMEM containing 2% horse serum for 5 days. All cells were cultured in a humidified atmosphere containing 5% CO2. For infection, 10^4^ cells were seeded in each well of a 24-well plate before infection with lentiviral vectors at a multiplicity of infection (MOI) of 10. Doxycycline was used at a final concentration of 1µg/ml.

### Immunofluorescence Analysis

Huh7 cells grown in Lab-Tek chamber slides, were fixed in 2% paraformaldehyde-PBS for 10 min, then permeabilized with 0.1% Triton X-100 for 45 min at room temperature. Fixed cells were incubated with 2 µg/ml anti-Tet-repressor (Mobitec; TET01) and then with a secondary Texas-red-conjugated anti-rabbit IgG (Becton Dickinson (BD). Stained cells were then mounted in ProLong mounting medium and observed using a fluorescence microscope.

### MicroRNA Quantification

miRNAs were extracted using mirVana miRNA isolation kit (Ambion, Applied Biosystems, Austin, TX) according to the manufacturer’s instructions. Mature miRNAs were measured by reverse transcriptase qPCR (RT-qPCR) using TaqMan miRNA assays (Applied Biosystems). In brief, 10 ng of total RNA was reverse transcribed with stem-loop RT primers specific for human miR-133a, miR-142.3p, or miR-122 (Applied Biosystems) using the TaqMan miRNA reverse transcription kit according to the manufacturer’s instructions. qPCR was performed on the resulting complementary DNA using miR-133a, miR-142 or miR-122 specific TaqMan primers and TaqMan universal PCR master mix in a 7900 real-time PCR instrument. The reactions were incubated in a 96-well optical plate at 95°C for 10 minutes, followed by 40 cycles of 95°C for 15 seconds and 60°C for 1 minute. Expression of the U6 gene was used as an endogenous control for data normalization. Stem-loop RT primer and TaqMan primer specific for human U6 were obtained from Applied Biosystems (Austin, TX). The amount of RNA from each sample was calibrated to the concentration of the small nucleolar RNA RNU6 (house-keeping gene). This then gave a delta CT (ΔCT) value for each miRNA (miRNA CT value – RNU6 CT value). The relative fold changes of miRNA was normalized to 293T cells. The fold difference in expression was calculated as 2^−(ΔCT sample−ΔCT in sample 293T)^.

### Total RNA Quantification

Total RNA was processed with Trizol as lysis buffer then extracted with the RNeasy Mini kit (Qiagen) according to the manufacturer’s instructions. For RT-qPCR, total RNA was initially reverse transcribed using the SuperScript III Platinum Two-Step kit (Invitrogen). Analysis of gene expression was carried out using the MESA GREEN qPCR MasterMix Plus for SYBR Assay (Eurogentec) and the TaqMan 7900 instrument. Total cDNA was added to a solution containing the primers and the Sybr Green PCR Master Mix, then loaded into a 96-well plate. Temperature cycling conditions were: 10 min at 95°C for activation followed by 40 cycles of 95°C for 15 s and 60°C for 1 min. The values were normalized to those obtained for the 18S gene. The results of the RT-qPCR are expressed as the Ct value for each sample. The sequences of the forward and reverse primers were the following. GFP F: 5′- ACT ACA ACA GCC ACA ACG TCT ATA TCA -3′ and GFP R: 5′- GGC GGA TCT TGA AGT TCA CC - 3′; 18S F: 5′ CCC TGC CCT TTG TAC ACA CC -3′ and 18S R: 5′- CGA TCC GAG GGC CTC ACT A -3′.

### FACS Analysis

Ten days after infection, transduced cells were analysed for GFP expression by FACS. Analysis was performed on an LSRII instrument (Becton Dickinson). Data were analysed with FlowJo software.

### Statistical Analysis

All results were compared statistically using unpaired two-tailed Student’s t-test with GraphPad Prism software. A p value of <0.05 was considered significant.

## Results

### Rationale

**Figure 1:**
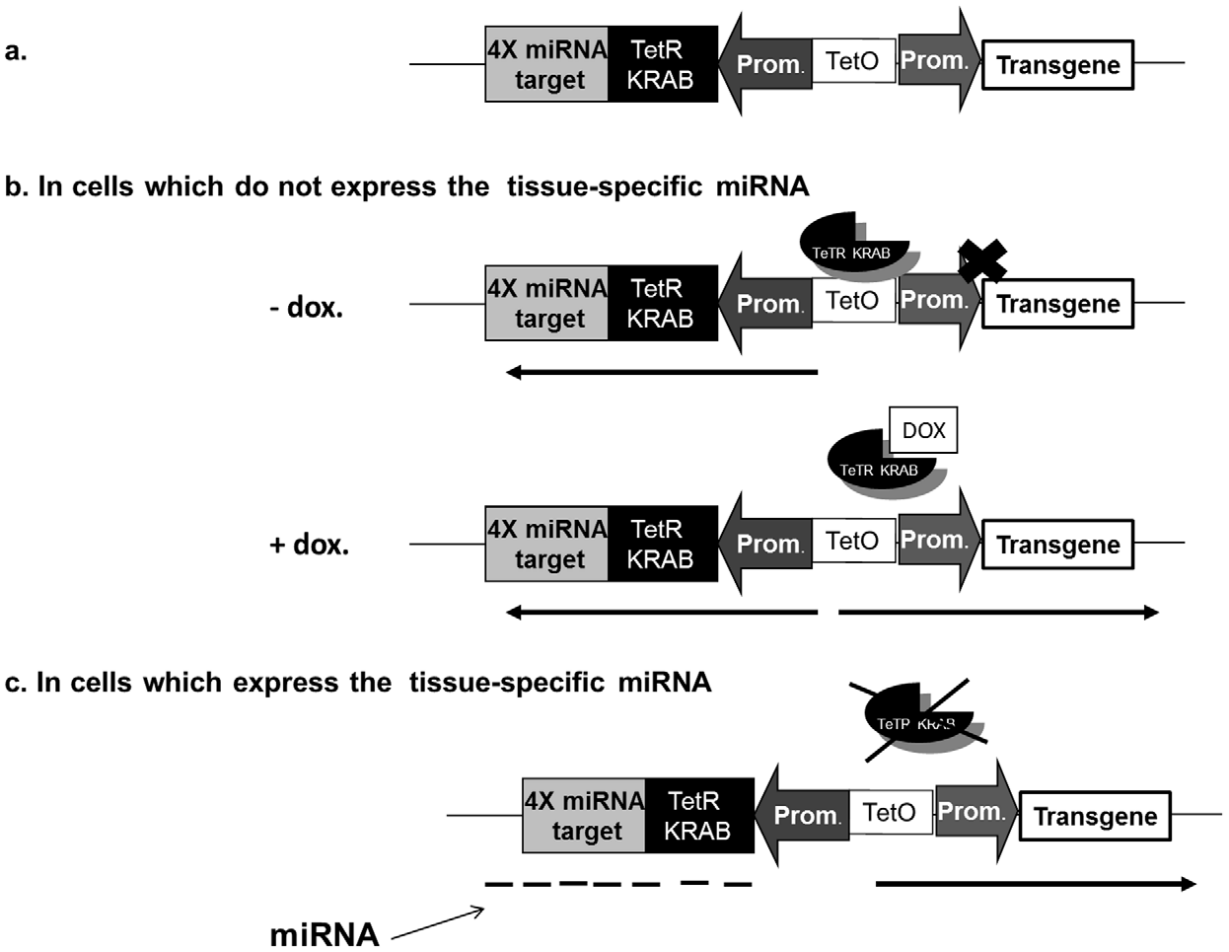
miRNA-based regulation of TetR-KRAB control of transgene expression. (**a**) Schematic representation of the miRNA-TetR-KRAB regulatory and reporter cassettes. (**b**) In the absence of tissue-specific miRNA, the repressor is translated and is free to bind the tetO operator, thus preventing transgene expression. However in the presence of a tetracyclin analog (Doxycycline) the repressor does not bind and transcription is activated.(**c**) In targeted cells tissue-specific miRNA binds to its complementary target and results in degradation of the TetR-KRAB mRNA. As a result, transgene expression occurs.

As shown in Figure 1a, our system comprises two different expression cassettes. The reporting cassette contains a marker or a transgene under control of a ubiquitously active or a liver specific promoter that has *tetO* binding sites. The second cassette is regulatory and encodes a TetR-KRAB–fusion protein with tissue-specific miRNA targets placed in the 3′ untranslated region of the transcript. In cells which do not express the tissue-specific miRNA (Fig. 1b) and in the absence of doxycycline, the transgene is not expressed. However the transgene can be expressed in the presence of doxycycline when binding of TetR-KRAB to the *tetO* sequences is prevented. In contrast, in cells expressing the tissue-specific miRNAs with cognates in the regulatory transcript, synthesis of the chimeric repressor is inhibited (Fig. 1c) and the transgene is then expressed. Therefore transgene expression is restricted to a specific target tissue, except in the presence of doxycycline when the binding of TetR-KRAB to the *tetO* sequences is prevented in non-target cells.

### In vitro Proof of Concept

**Figure 2:**
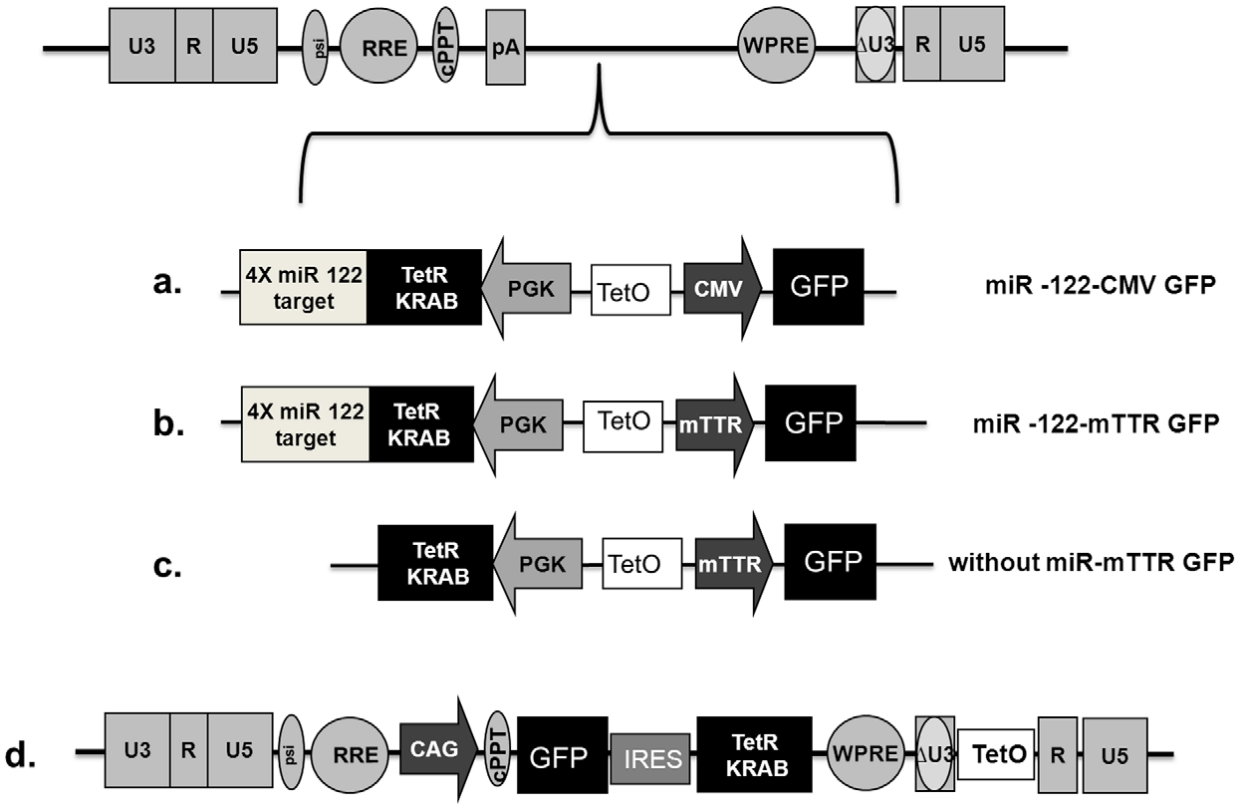
Schematic diagram of the recombinant lentivirus vectors used to accomplish liver-specific transgene expression. (**a**) **&** (**b**) Lentiviral vector encoding four target sequences of miR-122, TetR-KRAB and GFP under the control of a mTTR liver specific promoter (**a**) or constitutively active CMV promoter (**b**). (**c**) Lentiviral vector without target sequences of miR-122 and expressing GFP from liver-specific mTTR promoter. (**d**). Inducible TetR-KRAB lentiviral vector system that includes a GFP gene under control of a CAG promoter as part of a bicistronic unit comprising the KRAB based repressor (PLVCT). cPPT, central polypurine tract; IRES, internal ribosomal entry site; WPRE, woodchuck hepatitis virus post-transcriptional element; RRE: Rev protein responsive element; pA, polyadenylation site; CMV, cytomegalovirus; CAG, CMV immediate enhancer/β-actin; PGK, phosphoglycerate kinase; mTTR,murine liver-specific transthyretin receptor promoter.

**Figure 3:**
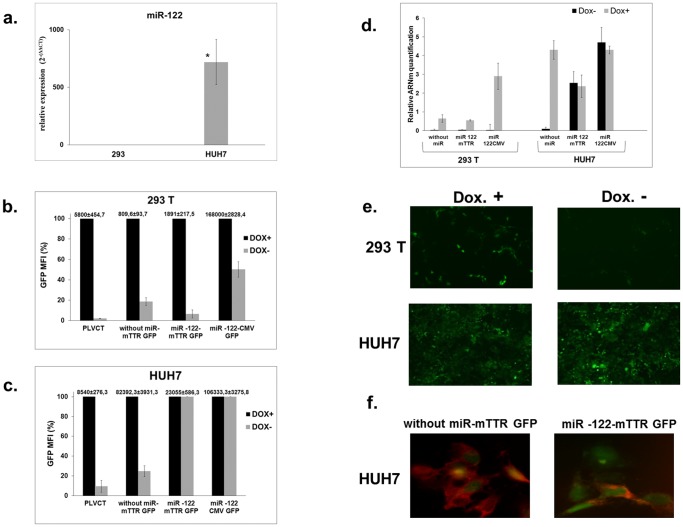
miR-122 concentrations and reporter gene expression in transduced cells. (**a**) miR-122 expression levels in Huh7 and 293T cells detected by RT-qPCR. Three independent experiments were performed in duplicate. *Statistically significant differences *P* = 0.03 (two-tailed, unpaired Student’s *t*-test). (**b**) **&** (**c**) GFP expression in transduced Huh7 and 293T cells. After 10 days of culture, with or without doxycycline, transduced 293T (**b**) or Huh7 (**c**) cells were analyzed for GFP expression by FACS. Results are expressed as a percentage of MFI. In the TetR-KRAB system, the expression of GFP is optimal in the presence of doxycycline and corresponds to 100% of the MFI. The raw data corresponding to 100% are indicated above each histogram bar. (**d**) RT-qPCR of GFP mRNA normalized to values obtained for 18S RNA. Data shown are mean and error bars indicate the standard deviation (SD) of 3 independent experiments performed in triplicate. (**e**) Representative images of GFP expression in cells at 10 days after transduction. Lentiviral vectors encoding TetR-KRAB mRNA with four target sequences for miR-122 and GFP gene under the control of a liver specific promoter in 293T and Huh7 cells with or without doxycycline treatment. Magnifications ×50. (**f**) Representative high power fields showing immunofluorescence staining of TetR-KRAB (red) in Huh7 cells transduced with recombinant lentiviruses. Four target sequences for miR-122 were absent (left panel) or present (right panel) in the TetR-KRAB-expressing cassettes of the vectors, which also produced GFP (green) constitutively. Magnifications ×400.

In a first set of experiments, we evaluated the efficiency of the system in liver-derived cells. We designed a regulatory cassette in which four copies of the miR-122 target sequence were inserted immediately downstream of the TetR-KRAB coding region. The targets were perfectly complementary to miR-122, which is specifically expressed in cells of hepatic lineage [17]. In the reporting cassette, green fluorescent protein (GFP) was inserted downstream of a cytomegalovirus (CMV) promoter (Fig. 2a) or of the liver-specific murine transthyretin receptor (mTTR) promoter (Figs. 2b, c). The cassettes were inserted in a lentiviral backbone to generate lentiviral vectors that are suitable for in vitro characterisation. As a control, we used a PLVCT vector (Fig. 2d) in which the TetR-KRAB cDNA is expressed from the ubiquitously active CAG (CMV immediate enhancer/β-actin) -promoter as part of a bicistronic transcript that also expresses GFP. We used Huh7 cells of hepatic origin, known to express miR-122, and 293T cells derived from human embryonic kidney, which do not produce miR-122 (Fig. 3a). Huh7 and 293T cells were transduced with the different vectors at a multiplicity of infection (MOI) of 10. The proportion of GFP-positive cells in the presence of doxycycline ranged between 24% and 98%. GFP expression was quantified by fluorescence-activated cell sorter (FACS) analysis and we compared GFP expression in the presence or absence of doxycycline in each cell line (Figs. 3b, c). After transduction with PLVCT, we observed very tight regulation of GFP expression. Indeed, the residual GFP mean fluorescence intensity (MFI) in the absence of doxycyline, is about 2% in 293T cells, and 10% in Huh7 cells. Using our TetR-KRAB regulation system without miRNA target sequences, in 293T cells we observed an 82% decrease in GFP MFI when doxycycline was withdrawn (Fig. 3b). This demonstrated the expected decrease in GFP expression effected by the TetR-KRAB protein in the absence of doxycycline. Compared to reporter gene expression from cassettes containing the constitutively active CMV promoter, decreased GFP expression was more efficient when GFP was under the control of the liver*-*specific mTTR promoter. As shown in Figure 3b, using constructs that contain ubiquitous promoters driving GFP, there was a decrease in GFP MFI of about 50% in 293 T cells. However, with the mTTR promoter the decrease ranged from 82% to 94% (Figs. 3b, e). In Huh7 cells incubated in the absence of doxycycline, we observed a decrease in MFI when lentiviral constructs without the miR-122 target sequences were used (76 to 90%). Immunofluorescence confirmed the presence of TetR-KRAB protein (Fig. 3f) in these cells. In contrast, when vectors containing the miR-122 target sequence were used, cells expressed little or no TetR-KRAB protein (Fig. 3f), and there was no change in GFP expression with or without doxycycline (Figs. 3c, e). These results were confirmed by quantification of GFP mRNA in the presence or absence of doxycycline in each cell line transduced by vectors expressing the different TetR-KRAB cassettes (Fig. 3d). In 293T cells incubated in the absence of doxycycline, we observed a very significant decrease in GFP mRNA levels with all constructs (more than 90%). In contrast, in Huh7 cells GFP mRNA levels were unaltered with lentiviral constructs carrying the miR-122 target sequences. These results therefore demonstrate well regulated transgene expression when miR-122 target sequences are included in the TetR-KRAB-encoding transcript.

**Figure 4:**
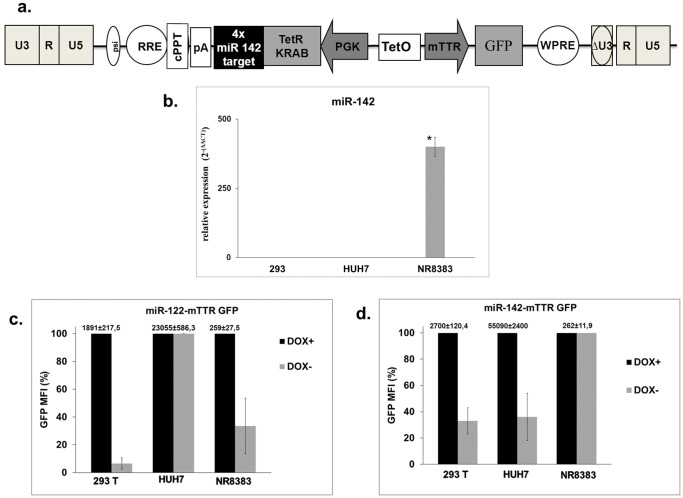
Lentiviral vectors used to target transgene expression to macrophage-derived cells. (**a**) Schematic diagram of the lentivirus vectors used to target cells of immune lineage. (**b**) miR-142 expression levels in Huh7, 293T and NR8383 cells detected by RT-qPCR. Three independent experiments were performed in duplicate. * Statistically significant differences *P*  = 0.043 (two-tailed, unpaired Student’s *t*-test). (**c**) **&** (**d**) Huh7, 293T and NR8383 cells were transduced with two different lentiviral vectors carrying the TetR-KRAB sequence follow by 4 copies of miR-142 target (c) or miR-122 (d). After 10 days of culture with or without doxycycline, transduced cells were analyzed for GFP expression by FACS. Results are expressed as a percentage of mean fluorescence intensity (MFI). In TetR-Krab system, the expression of GFP is optimal in the presence of doxycycline, corresponding to 100% of the MFI. The raw data corresponding to 100% are indicated above each histogram bar.Data shown are mean and error bars indicate the SD of 3 independent experiments performed in triplicate.

We subsequently extended our study to other tissues where specific transgene expression may be important in the context of gene therapy. We designed a DNA cassette in which the TetR-KRAB coding sequence contained 4 copies of perfectly complementary miR-142 target sequences. This miRNA is specifically expressed in cells from the immune system (Fig. 4a). As a positive target we used rat alveolar macrophage-derived cells (NR8383 line). Expected specific expression of miR-142 in NR8383 and its absence in Huh7 and 293T cells was demonstrated using RT-qPCR (Fig. 4b). To analyse regulation of transgene expression, NR8383, 293T and Huh7 cells were transduced at a MOI of 10 with a lentiviral vector carrying either miR-142 or miR-122 target sequences. We compared GFP expression in the presence or absence of doxycycline in each cell line (Figs. 4c, d). In 293T cells we observed a significant decrease in mean fluorescence intensity when doxycycline was withdrawn from cells that had been infected with vectors containing miR-142-mTTR GFP and miR-122-mTTR GFP cassettes. In Huh7 cells, as shown above, there was no variation in GFP expression when doxycycline was withdrawn after infection with miR-122 target sequence-containing vectors. In contrast a significant decrease was detected with vectors containing the miR-142 target sequences. In NR8383 cells there was a 70% decrease of GFP expression after doxycycline withdrawal when cells were transduced with miR-122 regulated vectors. After transduction with miR-142-regulated vectors, transgene expression did not change in the absence or presence of doxycycline (Figs. 4c, d). This indicated that the miR-142 target sequence enabled specific regulation in cells of the hematopoietic lineage.

**Figure 5:**
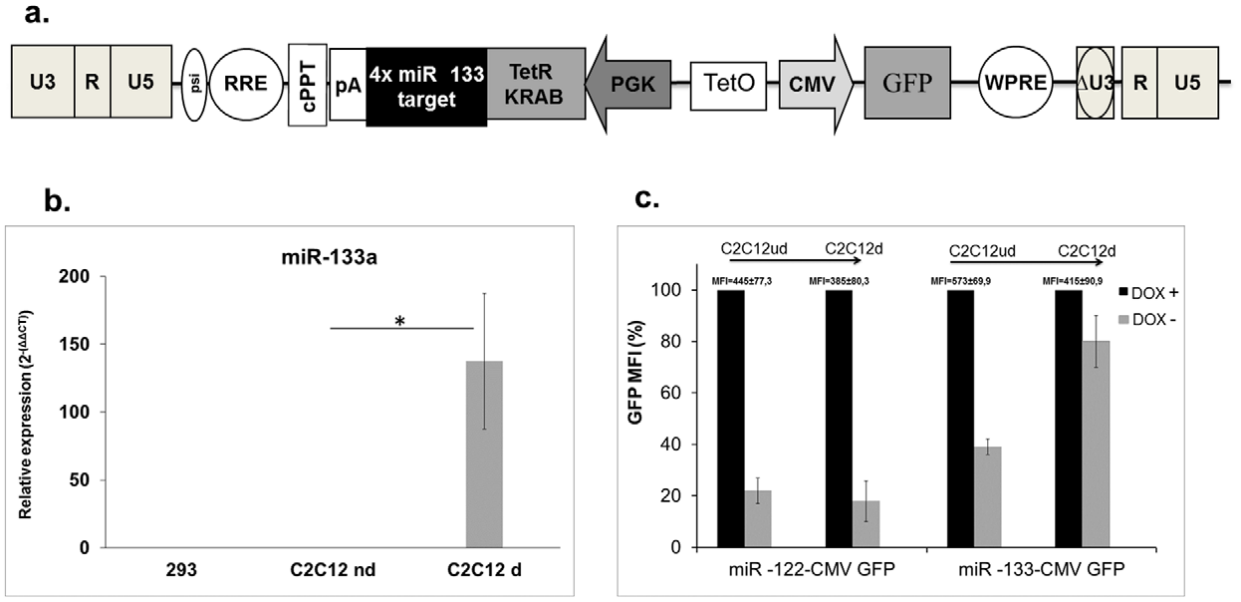
Lentiviral vectors used to target transgene expression to skeletal muscle-derived cells. (**a**) Schematic diagram of the lentivirus vectors used to accomplish skeletal muscle-specific expression. (**b**) miR-133 expression levels in C2C12d, C2C12ud and 293T cells detected by RT-qPCR. Three independent experiments were performed in duplicate. *Statistically significant differences *P* = 0.0185 (two-tailed, unpaired Student’s *t*-test). (**c**) Differentiated or undifferentiated C2C12 cells (C2C12d and C2C12ud respectively) were transduced with lentiviral vectors carrying the TetR-KRAB sequence followed by four copies of miRNA target of miR-122 or miR-133. After 10 days of culture with or without doxycycline, transduced cells were analyzed for GFP expression by FACS. Results are expressed as a percent of MFI. The raw data corresponding to 100% are indicated above each histogram bar. Data shown are mean and error bars indicate the SD of 3 independent experiments performed in triplicate.

We evaluated the efficiency of the system for regulating gene expression in muscle. To this end we designed lentiviral vectors containing four copies of perfectly complementary miR-133 targets downstream of the Tet-KRAB-encoding sequence. This miRNA is specifically expressed in adult cardiac and skeletal muscle tissues (Fig. 5a). We used C2C12 myoblasts because these cells are able to mimic skeletal muscle differentiation in vitro. We confirmed that expression of miR-133 increased when C2C12 cells underwent myoblast differentiation (Fig. 5b). Differentiated or undifferentiated C2C12 cells were transduced at a MOI of 10 with miR-133-CMV-GFP regulated or miR-122-CMV-GFP regulated lentiviral vectors. The expression of GFP was quantified by FACS analysis and we compared expression of the reporter in the presence or absence of doxycycline (Fig. 5c). GFP expression did not change during differentiation in cells transduced with the lentiviral vector carrying miR-122 target sequences. Moreover, doxycycline withdrawal caused a similar decrease in GFP expression in differentiated and undifferentiated cells. In contrast, in cells infected with the lentiviral vector carrying miR-133 target sequences we observed a significant reduction of GFP expression in undifferentiated cells after doxycyline removal, indicating that the TetR-KRAB regulation functioned according to the intended design. However, after differentiation, TetR-KRAB inhibition of GFP expression was severely impaired, indicating that the miR-133 expression silenced TetR-KRAB production. A slight decrease in GFP expression persists in the differentiated cells, which is likely to be a result of a small number of undifferentiated cells expressing little or no miR-133.

### Efficient miRNA-mediated TetR-KRAB Regulation in vivo

**Figure 6:**
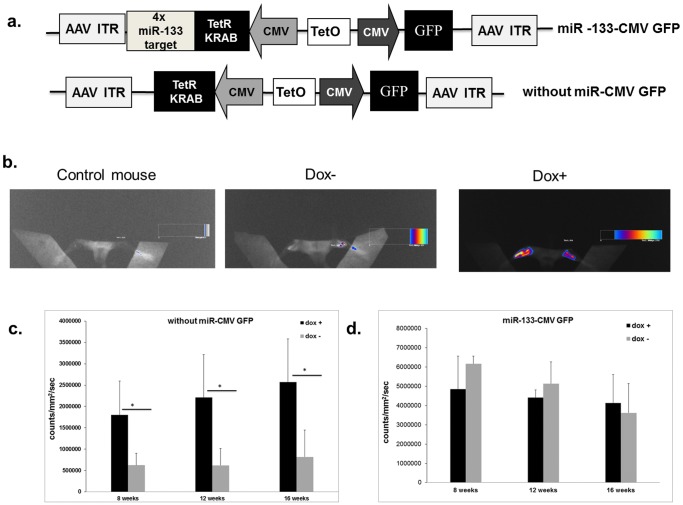
Assessment of efficacy of miRNA-regulated TetR-KRAB control of transgene expression in vivo. (**a**) Schematic diagram of the AAV vectors used for in vivo studies. (**b**) GFP ﬂuorescence was measured in live animals at 8 weeks after injection with recombinant AAVs. Mice received AAVs encoding the TetR-KRAB sequence without copies of miRNA target located downstream. Animals did or did not receive doxycycline in their drinking water. The color scale next to the images indicates the signal intensity. (**c**) **&** (**d**) Results at 8, 12 and 16 weeks post injection are expressed in counts/mm^2^/second. Data shown are mean and error bars indicate the SD. * Statistically significant differences *P*<0.05 (two-tailed, unpaired Student’s *t*-test), n = 4 per group.

To test the effectiveness of our system in vivo, the cassettes were inserted into recombinant adeno-associated virus (AAV) 2/8 vectors. We designed two DNA cassettes containing the TetR-KRAB coding sequence with four copies of miR-133 or without miRNA target sequences located in the downstream untranslated region. The GFP- and TetR-KRAB-coding sequences were under the control of a CMV promoter. These vectors were injected into the two tibialis anterior muscles of subject mice (n = 8 for each vector). At 2 weeks after AAV injection, three animals from each group received doxycycline in their drinking water. Mice were analyzed for GFP expression using fluorescence imaging 8 weeks after injection (Fig. 6b). GFP expression in muscle was scored and we compared GFP expression in the muscle in the presence or absence of doxycycline with each vector (Fig. 6c, d). In mice injected with AAV constructs without miRtarget sequences, we observed a strong fluorescence signal in muscle when animals received doxycycline. In contrast, in the absence of doxycycline, there was a sharp (about 70%) decrease in fluorescence (Fig. 6b, c). However, in mice injected with AAV carrying miR-133 target sequences, there was no variation of GFP expression in the absence or presence of doxycycline (Fig. 6d). The same animals were re-analyzed at 12 and 16 weeks after injection. The intended regulation by doxycyline persisted in muscles injected with AAV that did not contain the miRNA target sequences although no significant effect of doxycycline was recorded in mice injected with the miR-133-regulated vector (Fig. 6c, d). We have verified that miR-133-CMV-GFP regulated AAV vectors was efficient in liver in which the endogenous miR is absent. AAV carrying miR-133 target sequences were injected in the penile vein of mice (n = 8). At 2 weeks after AAV injection, half of animals received doxycycline in their drinking water. Mice were sacrificed 8 weeks after injection and GFP expression in liver was analysed by RT-PCR. We observed a significant increase in GFP expression in doxycycline-treated mice (**2^−ΔΔ^** ***C*** **_T_ = **16.7**,** data not shown).

## Discussion

Many natural or artificial promoters, with activity that is restricted to specific tissues, have been used in gene transfer vectors to achieve targeted expression of a transgene. However, it has been shown that these promoters are usually weak when compared to their constitutively active counterparts. Moreover these promoters are often leaky, which compromises their tissue specificity. Consequently an additional layer of control is required to achieve robust transgene transcription and limit expression to intended target cells. To address this, we have used tissue-specific expression of miRNAs to silence a transcriptional repressor. We have designed an original new system which combines use of the tetR-KRAB artificial transgene-repressor with endogenous miRNA silencing machinery. Specific and high level expression in targeted-tissue could be achieved, and this was independent of the promoter that was used. Expression of the TetR-KRAB component is under control of miR target sequences that were selected according to the miR expression profile of specific cell types. In our design, translation of the TetR-KRAB repressor is shut off in permissive cells that express the miRNAs, which in turn results in de-repression of the transgene. This system is versatile, and we obtained targeted expression in several cell types by including selected miRNA targets downstream of the TetR-KRAB-encoding sequences. Muscle-specific miR-133, liver specific miR-122, or hematopoietic specific miR-142 target sequences were shown to work synergistically with the TetR-KRAB cassette and enable tissue-specific expression.

An additional advantage of the regulatory system we describe is that the size of the paired cassettes is small enough to be inserted into most vectors that are currently used for gene transfer. In the study reported here, we successfully used lentiviral and AAV vectors to demonstrate utility of the system. We are currently investigating the use of alternate vectors, such as adenoviral and integration-defective lentiviral vectors, to effect TetR-KRAB silencing [18], [19].

In cells that do not express the tissue-specific miR, we observed a decrease in reporter transgene expression (between 50 and 94%), which was demonstrated in vivo and in vitro. However this effect was influenced by the promoters and is cell type-dependent. The decrease was measured as the ratio of transgene expression in the presence and absence of doxycycline. Complete shut-off of the transgene was however not achieved and could be a result of transcriptional interference between the two promoters. Indeed, viral vectors containing two transcription units in tandem have frequently been reported to be compromised by mutual promoter suppression [20], [21]. However, when two promoters are fused back to back, interference between them has been reported to be minimal [22], [23]. A critical parameter that may influence the efficiency of the system we developed is the location of the *tetO* sequence. In addition to the configuration described here, we designed a lentiviral vector in which the *tetO* sequence was inserted into the U3 region of the 3′ LTR (data not shown). During reverse transcription, the 3′ U3 RNA region serves as the template for synthesis of its 5′ DNA homologue and the *tet*O sequence is duplicated in the integrated provirus. We found that this arrangement was less efficient than the configuration described here, suggesting that duplicating *tetO* sequences does not improve effectiveness of transgene repression. We also compared our system to a previously described inducible TetR-KRAB system (PLVCT) [14]. As with the results reported by Szulc and colleagues, we also observed a decrease in GFP expression in 293T cells when incubated in the absence of doxycycline. However we found that the efficiency varied in different cell types. After doxycycline withdrawal, there was a more pronounced decrease in GFP expression compared to cells of hematopoietic origin. Therefore our results indicate that in cells which do not express the tissue-specific miRNA, the decrease of transgene expression may vary. In addition, the promoter type may influence the doxycycline responses. Nevertheless, our system is still efficient and allows for improvement in the tissue specificity by significantly reducing background transgene expression.

Preventing cytotoxic immune responses to transgene products is important for gene therapy, and the system described here provides the means for achieving this. We and others have previously documented that an immune response to a transgene product can impair the desired long term expression following hepatic transgene delivery when using oncoretroviral, AAV or lentiviral vectors [24], [25], [26], [27], [28], [29]. Frequently, epitopes of the transgene product are presented by antigen presenting cells, resulting in an undesirable immune response and premature removal of the transgene. One of the approaches to overcome initiation of a vigorous immune response is to prevent transgene expression in antigen presenting cells, and using lineage-specific miRNAs to inhibit transgene expression allows for this. Brown and colleagues used target sequences of hematopoietic lineage-specific miR-142.3 to eliminate off-target expression in hematopoietic cells. Although this strategy accomplished sustained gene transfer in haemophilia B mice for more than 280 days [30], the method was insufficient to prevent an anti-FVIII immune response in treated haemophilia A mice [31]. We believe that our approach improves gene transfer efficiency by allowing high expression in targeted cells, but at the same time limiting expression in non-targeted cells, especially those of hematopoietic origin.

In addition to transgene-directed immunity, development of an immune response against the exogenous trans-activators or trans-repressors has been reported in nonhuman primates [32], [33]. We believe that this may not compromise efficiency of transgene tissue targeting of the system we described here. If an immune response to the TetR-KRAB chimeric protein develops, it should be directed to non-targeted cells that express the TetR-KRAB protein, while targeted cells that do not express the TetR-KRAB should be spared. In conclusion, we believe that the system we have developed and described here is original and versatile. It can be exploited to achieve specific and robust transgene expression that is critical in successfully developing gene therapy.
